# Complement effectors, C5a and C3a, in cystic fibrosis lung fluid correlate with disease severity

**DOI:** 10.1371/journal.pone.0173257

**Published:** 2017-03-09

**Authors:** Pamela S. Hair, Laura A. Sass, Turaj Vazifedan, Tushar A. Shah, Neel K. Krishna, Kenji M. Cunnion

**Affiliations:** 1 Department of Pediatrics, Eastern Virginia Medical School, Norfolk, Virginia, United States of America; 2 Children's Specialty Group, Norfolk, Virginia, United States of America; 3 Children’s Hospital of The King’s Daughters, Norfolk, Virginia, United States of America; 4 Department of Microbiology and Molecular Cell Biology, Eastern Virginia Medical School, Norfolk, Virginia, United States of America; Laurentian, CANADA

## Abstract

In cystic fibrosis (CF), lung damage is mediated by a cycle of obstruction, infection, inflammation and tissue destruction. The complement system is a major mediator of inflammation for many diseases with the effectors C5a and C3a often playing important roles. We have previously shown in a small pilot study that CF sputum soluble fraction concentrations of C5a and C3a were associated with clinical measures of CF disease. Here we report a much larger study of 34 CF subjects providing 169 testable sputum samples allowing longitudinal evaluation comparing C5a and C3a with clinical markers. Levels of the strongly pro-inflammatory C5a correlated negatively with FEV1% predicted (P < 0.001), whereas the often anti-inflammatory C3a correlated positively with FEV1% predicted (P = 0.01). C5a concentrations correlated negatively with BMI percentile (P = 0.017), positively with worsening of an acute pulmonary exacerbation score (P = 0.007) and positively with *P*. *aeruginosa* growth in sputum (P = 0.002). C5a levels also correlated positively with concentrations of other sputum markers associated with worse CF lung disease including neutrophil elastase (P < 0.001), myeloperoxidase activity (P = 0.006) and DNA concentration (P < 0.001). In contrast to C5a, C3a levels correlated negatively with worse acute pulmonary exacerbation score and correlated negatively with sputum concentrations of neutrophil elastase, myeloperoxidase activity and DNA concentration. In summary, these data suggest that in CF sputum, increased C5a is associated with increased inflammation and poorer clinical measures, whereas increased C3a appears to be associated with less inflammation and improved clinical measures.

## Introduction

Cystic fibrosis (CF) afflicts more than 70,000 individuals worldwide with respiratory failure causing the majority of deaths. CF lung disease is mediated by a cycle of obstruction, infection with microbial pathogens, and inflammation [1] leading to progressive lung tissue damage, scarring and finally pulmonary failure.

In humans, the most destructive inflammatory cascade is the complement system, which contributes to numerous inflammatory disease processes [2]. Proteomic analysis revealed that complement proteins C3 and C4 are major constituents of CF lung fluid [3]. Two of the most important effectors generated by complement activation include C5a and C3a [4]. C5a powerfully stimulates neutrophil migration and activation, leading to oxidative burst and degranulation [5, 6]. Neutrophil death in the lungs is a major source of viscous DNA worsening airway obstruction [7, 8]. Neutrophil elastase is a destructive enzyme that strongly contributes to lung damage [9–11]. C5a also stimulates histamine release, increases vascular permeability, and smooth muscle contraction [5]. C5a is increasingly appreciated for contributing to inflammatory lung diseases [12, 13] and acute lung injury [13]. Fick et al [14] demonstrated increased amounts of C5a in the bronchoalveolar lavage (BAL) of 9 CF patients with stable lung disease compared with healthy controls. Two (2) CF patients with the lowest C5a measurements had normal FEV_1_ and FVC measurements.

In contrast to C5a, several studies have shown that C3a may be anti-inflammatory, especially in the acute phase of the disease process by limiting mobilization and accumulation of neutrophils in tissues. Thus, in an acute exacerbation of a chronic disease, C3a may act in direct opposition to the pro-inflammatory C5a [15, 16].

In our previous pilot study we evaluated soluble fractions (sols) from sputum samples of 15 CF patients, each providing a single sample, for complement effectors in concert with clinical measurements [17]. The pro-inflammatory peptide C5a was increased nearly 5-fold in CF sols compared with controls. In children, C5a in CF sol correlated inversely with body mass index (BMI) percentile. C3a correlated positively with FEV1% predicted. Here we report a large, longitudinal study of CF patients that demonstrate the correlation of sputum C5a and C3a with clinical measures of CF disease severity.

## Materials and methods

All relevant data are within the paper.

### Ethics statement

Sputum samples were obtained from consented patients as part of their standard of care visit at the Children’s Hospital of The King’s Daughters Cystic Fibrosis Center under an Eastern Virginia Medical School IRB approved protocol 12-08-EX-0200 with written consent obtained by Dr. Sass.

### Study design

This was a prospective longitudinal cohort of 35 CF subjects at the Children’s Hospital of The King’s Daughters Cystic Fibrosis Center. 200 sputum samples were obtained between February 8, 2013 and February 19, 2016. After rejecting samples yielding insufficient sols for the intended assays, 169 samples were tested. The number of sols tested per subject ranged from 1–13, with a mean of 5 sols per subject.

### Sputum sols

Expectorated sputum samples were placed immediately on ice. The soluble (sol) fraction was recovered after cold (4°C) centrifugation at 14,000 × *g* for 60 minutes, as previously described [18]. The free-flowing sol fractions were not normalized for protein content, consistent with previously described methods [19].

### Clinical data

Clinical data were obtained from the electronic and written medical record for the clinic visit at which the sputum sample was collected. The FEV1% predicted was obtained from pulmonary function testing performed the same day as sputum collection. The most recent radiograph prior to sputum collection was scored for bronchiectasis: 0 = normal; 1 = 1 lobe, mild; 2 = 2–4 lobes; 3 = all lobes [17]. Cystic fibrosis related diabetes (CFRD) score was based on the most recent endocrinology assessment prior to sputum collection: 0 = normal; 1 = glucose intolerance; 2 = CFRD [17]. A pulmonary exacerbation score (PES), adapted from Akron Children’s CF center [20], was calculated for each visit incorporating objective, subjective, and clinical information such that a score of ≥ 5 indicates an exacerbation. Organisms were recorded from the CF sputum culture performed in the clinical microbiology laboratory. The organisms were categorized as to whether the following were present or absent: *P*. *aeruginosa*, *S*. *aureus*, *B*. *cepacia* complex, or *Candida* species. Patient medications at the time of clinic visit were categorized as to whether the following were present or absent: systemic corticosteroid, inhaled corticosteroid, azithomycin, inhaled antibiotic, or systemic antibiotic (excluding azithromycin).

### ELISAs

The C5a and C3a concentrations in sputum sols were measured via ELISA kit (R&D Systems, Minneapolis, MN or BD Biosciences, San Jose, CA) [17, 21–23].

Elastase concentrations in sputum sols were measured via ELISA. A sheep anti-elastase antibody (Thermo Scientific) was coated onto an Immulon-2 ELISA plate in bicarbonate buffer overnight at 4°C. The plates were washed with PBST (phosphate buffered saline + 0.1% Tween) and then blocked with 3% BSA/PBS (bovine serum albumin in PBS) for 2 hours at room temperature. After washing, the plates were incubated with diluted samples or a standard curve made from pure elastase (Innovative Research) diluted in 3% BSA/PBS for 1 hour. After washing, the wells were probed with a mouse anti-elastase antibody (Thermo Scientific) followed by a goat anti-mouse HRP-labeled antibody (Sigma). Wells were developed using TMB substrate solution (Thermo Scientific), stopped using 1 N H_2_SO_4_, and read at 450 nm in a plate reader (BioTek).

MPO was quantitated by serially diluting the sputum sol in PBS in 0.02 ml aliquots until it gave a detectable absorbance reading (450 nm) when combined with 0.1 ml TMB. Purified neutrophils were used as an MPO source to establish a standard curve for the CF samples. The neutrophils were lysed with 2% Triton X-100, diluted to 6.25 × 10^6^ cell/ml, and then serially titrated in 0.02 ml aliquots and combined with 0.1 ml TMB to generate a standard curve. Using the linear regression of the standard curve along with the initial dilution of the CF sol, we calculated an MPO value equivalent to 10^6^ lysed PMN/ml.

DNA in sputum sol was assayed using Quant-it PicoGreen (Invitrogen). Briefly, the sol samples were serially diluted in Tris-EDTA buffer in 0.1 ml aliquots and combined with 0.1 ml of diluted PicoGreen reagent for five minutes in the dark. The fluorescence was then measured at 480 nm (excitation) and 520 nm (emission) on a spectrofluorometer (BioTek). Quantitation was calculated from a standard curve using pure DNA.

### Statistical analysis

Continuous variables were expressed as mean, standard deviation (SD), median, minimum and maximum, and categorical variables were expressed as proportions. Estimation of parameters (e.g., coefficient β), odds ratio and 95% Confidence Interval (C.I.) were obtained using Generalized Estimating Equations (GEE) model. The QIC (Quasi-likelihood under the Independence model Criterion) was used for model selection and finding an acceptable working correlation structure. A log-transformation was considered for some response variables. All statistical analyses were performed using RStudio Team (2015) and SPSS 23 (Chicago, IL, USA) for Windows 16.0. Statistical tests were two-tailed, and P value < 0.05 was considered statistically significant.

## Results

### Study subjects

The demographics of the 34 CF subjects at the time of enrollment are shown in Table 1. The mean age at enrollment was 24 years with a range of 2–66 years. Average FEV1% predicted at enrollment was 60% suggesting a population with moderate lung disease. Among the children (<20 years of age), mean BMI percentile was 41^st^ percentile, suggesting moderate nutritional insufficiency. Thirty-five percent began the study with a diagnosis of CFRD. At enrollment, 61% had sputum cultures that grew *S*. *aureus* and 48% had sputum cultures that grew *P*. *aeruginosa*.

**Table 1:** Demographics at study entry.

Variables	Legend	Value
**Study population**	N	34
**Age in years**	Mean ± SD	24.44 ± 14.07
**Gender**		
** Male**	Freq (%)	19 (55.9%)
** Female**	Freq (%)	15 (44.1%)
**FEV1% predicted**	Mean ± SD	0.60 ± 0.22
**BMI**		
** Age < 20 years (%ile)**	Mean ± SD	0.41 ± 0.21
** Age ≥ 20 years**	Mean ± SD	22.53 ± 3.27
**CFRD**		
** Yes**	Freq (%)	12 (35.3%)
** No**	Freq (%)	22 (64.7%)
***S*. *aureus***		
** Yes**	Freq (%)	19 (61.3%)
** No**	Freq (%)	12 (38.7%)
***P*. *aeruginosa***		
** Yes**	Freq (%)	15 (48.4%)
** No**	Freq (%)	16 (51.6%)

### Changes in C5a, C3a over time and age

**Fig 1:**
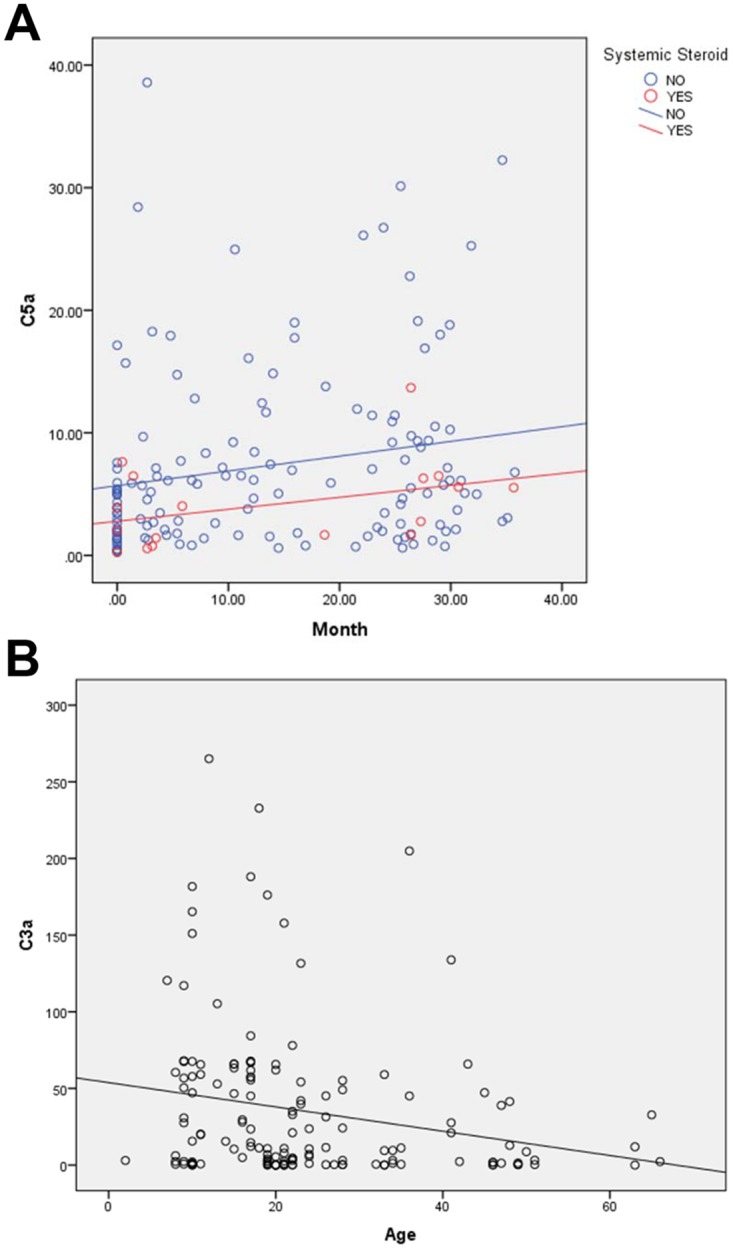
Changes in C5a and C3a over time or increasing age. (A) Changes in C5a (ng/ml) concentration over time and effect of systemic corticosteroid use. First sample for each subject is day zero. (B) C3a (ng/ml) compared with subjects age at specimen collection. Best fit line is shown for each graph.

Multivariate analysis using backward elimination demonstrated that sputum sol C5a concentrations increased over time (P = 0.028) (Fig 1A). Initial specimen for each subject is day zero. The level of C5a increased by 0.12 ng/ml per month with a 95% Confidence Interval (CI) of (0.01, 0.22). However, absolute age of the subject did not correlate with C5a level (P = 0.283). C5a concentration was lower by an average of 4.2 ng/ml (95% CI: 0.93, 7.49) for those taking a systemic steroid (P = 0.012). Sputum C3a concentrations correlated inversely with age (P = 0.021) (Fig 1B), but did not correlate with time after first sputum (P = 0.329). C3a level declined by 0.67 ng/ml per year (95% CI: 0.10, 1.23) as age increased. No other significant correlations with independent variables were identified for C5a and C3a. These results show that over the three years of the study sputum C5a levels increased over time and sputum C3a levels decreased with increasing age. This suggests that increasing C5a and decreasing C3a might be associated with CF disease progression.

### C5a and C3a correlation with FEV1% predicted and BMI percentile

**Fig 2:**
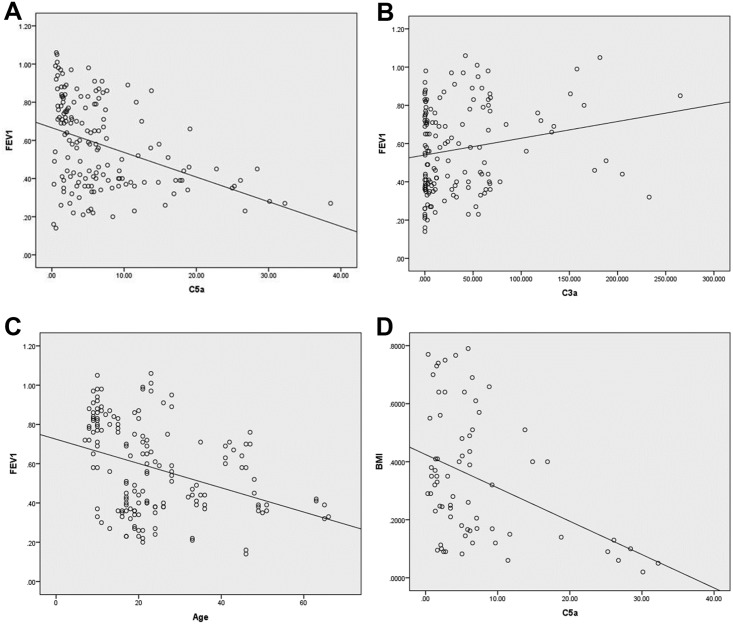
Correlation of C5a and C3a with FEV1% predicted and BMI percentile. (A) FEV1% predicted compared with C5a (ng/ml). (B) FEV1% predicted compared with C3a (ng/ml). (C) FEV1% predicted compared with age. (D) C5a concentration correlation with BMI percentile in ≤ 20 year olds. Best fit line is shown for each graph.

Multivariate analysis using backward elimination demonstrated that sputum C5a concentrations correlated negatively with FEV1% predicted (P < 0.001) (Fig 2A). FEV1% predicted decreased by 1 percentage point for each 1 ng/ml increase in C5a with a (95% CI: 0.8, 1.3). Sputum C3a concentrations correlated positively with FEV1% predicted (P = 0.01) (Fig 2B). FEV1% predicted increased 0.1 percentage point for each 1 ng/ml increase in C3a with a (95%CI: 0.0, 0.1). As expected, FEV1% predicted decreased with age (P < 0.001); a 0.6 percentage point decrease for each 1 year increase in age (95% CI: 0.3, 0.9) (Fig 2C). These results show a strong correlation between complement effectors and FEV1% predicted suggesting that C5a and C3a may have an impact on CF lung function.

Assessment of BMI was separated by age with ≤ 20 year old CF subjects measured as BMI percentile and adults measured as absolute BMI. The BMI percentile for children decreased over time (P = 0.017). C5a concentration correlated negatively with BMI percentile in children (P = 0.028) (Fig 2D). A C5a increase of 1 ng/ml decreased BMI percentile by 0.5 percentage points (95% CI: 0.1, 0.9). C3a concentration did not correlate with BMI percentile (P = 0.111). In adults, absolute BMI did not correlate with C5a (P = 0.253) nor C3a (P = 0.918). These findings demonstrate a negative correlation in children with CF between sputum C5a and BMI percentile, suggesting an association between elevated C5a level and increased disease severity.

### Longitudinal evaluation of C5a and FEV1% predicted

**Fig 3:**
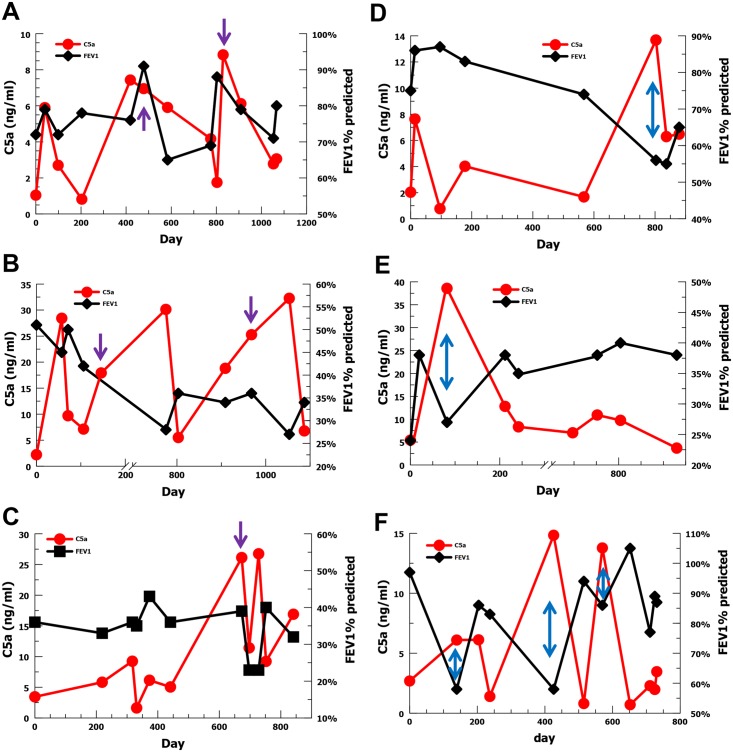
Longitudinal C5a level (red) and FEV1% predicted (black) for the six subjects with the most sputum samples. (A-C) For 3 subjects elevations in C5a concentration (purple arrows) appear to precede declines in FEV1% predicted. (D–F) For three subjects elevations in C5a concentration (blue arrows) appear to be coincident with declines in FEV1% predicted.

**Fig 4:**
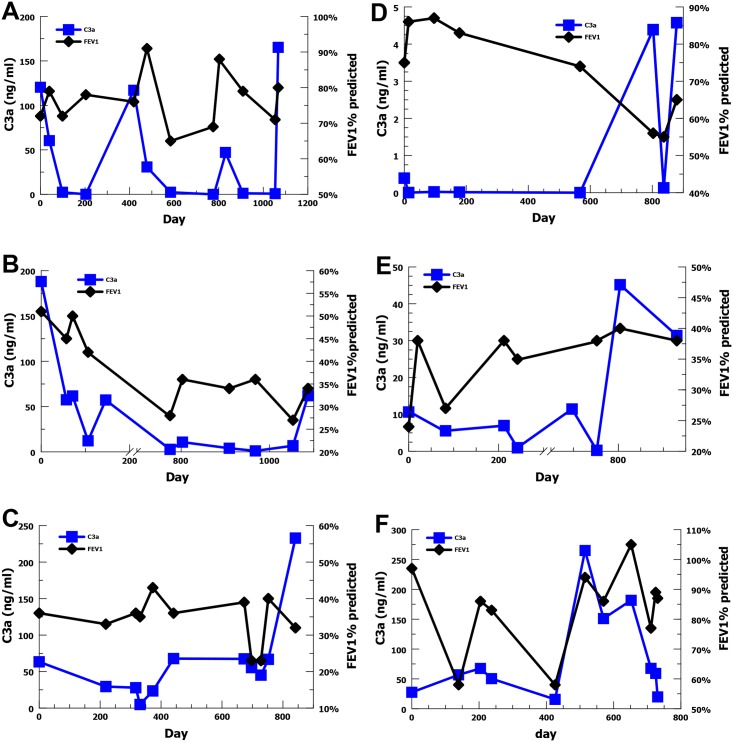
Longitudinal C3a level (blue) and FEV1% predicted (black) for the six subjects with the most sputum samples. (A-F) For 6 subjects changes in C3a do not appear to demonstrate a pattern with respect to acute changes in FEV1% predicted.

Given the association between increasing C5a level and decreased FEV1% predicted, we wanted to evaluate the temporal relationship for individual subjects with multiple sputum samples over the 3 years of the study. We chose the 6 subjects with the most sputum sample values ranging from 8–13 samples each. For three subjects, elevated or rising C5a concentrations appeared to precede declines in FEV1% predicted (Fig 3A–3C). For the other 3 subjects, elevated C5a concentrations appeared to be coincident with decreases in FEV1% predicted (Fig 3D–3F). These results suggest that generation of the C5a effector may precede or coincide with declines in FEV1% predicted providing further evidence that there may be a causal relationship. C3a concentrations were also evaluated longitudinally with FEV1% predicted for these 6 subjects (Fig 4A–4F), but no obvious association was identifiable with acute changes in FEV1% predicted at an individual level.

### C5a, C3a and other clinical indicators

Sputum C5a and C3a concentrations were correlated with an acute pulmonary exacerbation score (PES), degree of bronchiectasis, sputum positive for *S*. *aureus* and *P*. *aeruginosa*, and CFRD (Table 2). C5a level positively correlated with a PES ≥ 5, indicative of an acute worsening (P = 0.007). C3a level correlated negatively with a PES ≥ 5 (P < 0.001). C5a concentration correlated negatively with degree of bronchiectasis assessed radiographically (P < 0.001), but C3a level did not show a correlation (P = 0.899). *S*. *aureus* recovery from sputum culture did not correlate with C5a (P = 0.234) or C3a (P = 0.252). C5a level positively correlated with *P*. *aeruginosa* growth in sputum culture (P = 0.002), but C3a did not correlate (P = 0.187). Neither C5a nor C3a concentration correlated with having CRFD. Increased C5a correlation with acute pulmonary exacerbation is consistent with elevated C5a associated with decreased FEV1% predicted. C3a concentration negative correlation with PES is consistent with increased C3a associated with higher FEV1% predicted. *P*. *aeruginosa* growth in sputum associated with increased C5a further reinforces the association of C5a generation and CF lung disease.

**Table 2:** C5a and C3a correlations with Pulmonary Exacerbation Score (PES), bronchiectasis, *S*. *aureus*, *P*. *aeruginosa*, and Cystic Fibrosis Related Diabetes (CFRD).

	C5a sputum concentration			C3a sputum concentration		
	OR	95% CI	P	OR	95% CI	P
**PES ≥ 5**	1.08	1.021, 1.142	0.007	0.990	0.985, 0.995	<0.001
**Bronchiectasis**	0.816	0.764, 0.872	<0.001	1.000	0.996, 1.005	0.899
***S*. *aureus***	1.039	0.975, 1.108	0.234	0.996	0.989, 1.003	0.254
***P*. *aeruginosa***	1.102	1.037, 1.170	0.002	0.995	0.988, 1.002	0.187
**CFRD**	1.001	0.960, 1.044	0.954	0.995	0.987, 1.002	0.177

OR = Odds Ratio

### C5a, C3a and neutrophil mediators of CF lung disease

The potent ability of C5a to recruit neutrophils and stimulate degranulation [5, 24, 25], suggests that C5a could contribute to increased amounts of neutrophil mediators of lung disease in CF. C5a correlated positively with neutrophil elastase (NE) concentrations in CF sputum sols (P < 0.001) (Table 3). The concentration of NE increased 9.3 μg/ml for each 1 ng/ml increase in C5a (95% CI: 1.051, 1.136). C3a level inversely correlated with NE concentration (P < 0.001) such that NE decreased 1.2 μg/ml for each 1 ng/ml increase in C3a (95% CI: 0.984, 0.991). MPO functional activity was calculated using a standard curve generated from a lysate of 6.25 ×10^6^ neutrophils per ml. C5a concentration correlated positively with myeloperoxidase (MPO) concentration in CF sputum sol (P = 0.006) such that MPO increased 3 units (10^6^ PMN/ml) for each 1 ng/ml increase in C5a (95% CI: 1.009, 1.052). C3a correlated inversely with MPO (P = 0.002) where MPO decreased 0.4 units (10^6^ PMN/ml) for each 1 ng/ml increase in C3a (95% CI: 0.993, 0.998). C5a concentration correlated positively with DNA concentration in CF sputum sol (P < 0.001) such that DNA increased 3.6 μg/ml for each 1 ng/ml increase in C5a (95% CI: 1.020, 1.053). C3a concentration correlated inversely with DNA in sputum (P < 0.001) where DNA decreased 1 μg/ml for each 1ng/ml increase in C3a (95% CI: 0.987, 0.993). All three neutrophil mediated parameters correlated positively with increasing age: NE (P < 0.001), MPO (P < 0.001) and DNA (P < 0.001). Together, these data demonstrate strong correlations between C5a, C3a and neutrophil mediators of CF lung disease and are consistent with our findings for clinical parameters suggesting that elevated C5a correlates with worse lung disease and elevated C3a correlates with less lung disease.

**Table 3:** C5a and C3a correlations with sputum concentration of Neutrophil Elastase (NE), myeloperoxidase (MPO) and DNA.

	C5a sputum concentration			C3a sputum concentration		
	β	95% CI	P	β	95% CI	P
**NE**	1.093	1.051, 1.136	<0.001	0.998	0.984, 0.991	<0.001
**MPO**	1.030	1.009, 1.052	0.006	0.996	0.993, 0.998	0.002
**DNA**	1.036	1.020, 1.053	<0.001	0.990	0.987, 0.993	<0.001

β = coefficient beta

## Discussion

These data are unique in providing longitudinal data for complement effectors in CF sputum sols over a three year study period. Having 169 testable samples allowed for robust statistical modeling. Confidence in validity is enhanced by demonstrating the typical, although not ideal, trends of decreasing FEV1% predicted with increasing age and decreasing BMI percentile over time, consistent with progression of CF disease.

Our previous pilot study suggested the C5a concentration in CF sputum sol correlated inversely with BMI percentile and C3a concentration correlated positively with FEV1% predicted [17]. This much more robust dataset confirms and expands upon these associations. We now show that C5a has a strong negative correlation with FEV1% predicted. This finding is corroborated by the positive association of elevated C5a level with a PES score suggestive of an acute pulmonary exacerbation. The association of elevated C5a level with *P*. *aeruginosa* recovered from sputum culture and the strong positive correlation of C5a level with neutrophil mediators of CF lung disease, NE, MPO and DNA, provide additional supporting evidence. This raises the possibility that increases in C5a could contribute to declines in pulmonary function or pulmonary exacerbations could increase C5a generation. Longitudinal data for individual CF subjects with ≥ 8 testable specimens suggests that increases in C5a level generally either precede or are simultaneous with decreases in FEV1% predicted. Together these data suggest that increased C5a generation in the CF lung is more likely to be an inciting factor in acute CF lung disease, consistent with its known pro-inflammatory properties, rather than a down-stream reactor lagging behind the disease process.

In these data, C3a concentrations in CF sputum sols correlated positively with FEV1% predicted and inversely with acute pulmonary exacerbation score (PES), suggesting an anti-inflammatory role consistent with prior findings that C3a can inhibit neutrophil migration [16]. This is supported by increased C3a concentrations being associated with decreased concentrations of neutrophil effectors, NE, MPO and DNA.

In summary, these data demonstrate strong associations between complement effectors C5a and C3a in CF lung fluid and important clinical measures of CF disease severity and disease progression. It suggests that complement effectors may play important roles in CF lung inflammation. This raises the possibility that complement-modulation could be developed as a treatment modality for CF patients or C5a/C3a concentrations in CF sputum could be used as biomarkers. These findings warrant future studies testing complement modulation in animal models of CF.

## Abbreviations

BAL: Bronchoalveolar Lavage
BMI: Body Mass Index
CF: Cystic Fibrosis
CFRD: Cystic Fibrosis Related Diabetes
FEV1%predicted: Forced Expiratory Volume in 1 second, percent of predicted
MRSA: Methicillin-resistant Staphylococcus aureus
